# Zinc nanoparticles alleviate vanadium stress in wheat (*Triticum aestivum* L.): impacts on vanadium accumulation, nitrogen assimilation, and amino acid profile

**DOI:** 10.1186/s12870-026-09465-y

**Published:** 2026-07-13

**Authors:** Zhe Wang, Yongping Zhao, Chengjing Tang, Debao Yao, Yanlan Liu, Xue Mi, Yanhua Li

**Affiliations:** 1 School of Biomedical and Food Engineering, Shangluo College, North New Road, #10, Shangluo, 726000 Shannxi China; 2 Institute of Dryland Agriculture, Gansu Academy of Agricultural Sciences, LanzhouGansu, 730070 China; 3 College of Life Science, Northwest University, Xi’an, 710021 Shannxi China; 4 International College of Maejio University, Chiang Mai, 50290 Thailand

**Keywords:** Vanadium stress, Zinc oxide nanoparticles, Nitrogen assimilation, Grain yield, Amino acid profile

## Abstract

Zinc oxide nanoparticles (ZnO-NPs) have shown potential in mitigating vanadium (V) toxicity in plants, but their effects on V accumulation and grain nutritional quality remain unclear. To address this, we conducted a pot experiment using wheat treated with three levels of V (0, 100, and 200 mg/kg) and three levels of ZnO-NPs (0, 100, and 200 mg/kg), and systematically assessed the effects of ZnO-NPs on plant growth, V accumulation, nitrogen assimilation, and the seed amino acid profiles. Results showed that ZnO-NPs alleviated V stress by reducing V uptake and restoring growth. Compared to the control (without V and ZnO-NPs), 100 mg/kg V stress reduced total biomass and grain yield by 12.58% and 31.94%, respectively; co-application of 100 mg/kg ZnO-NPs reversed this decline, increasing total biomass and grain yield by 13.60% and 44.95% compared to V stress alone. Similarly, 100 mg/kg V stress decreased seed N, P, and K content by 8.63%, 19.12%, and 12.73%; co-application of 100 mg/kg ZnO-NPs significantly increased them by 11.64%, 22.01%, and 13.98%. Notably, the 100 mg/kg V stress also reduced the total amino acids, essential amino acids, and the essential amino acid index by 21.94%, 25.39%, and 7.12%, respectively, compared to the control; the co-application of 200 mg/kg ZnO-NPs significantly increased these indices by 21.25%, 32.06%, and 9.36%, respectively. Furthermore, under 100 mg/kg V stress, co-application of 200 mg/kg ZnO-NPs increased the valine, isoleucine, leucine, phenylalanine, histidine, and lysine contents by 12.55%, 12.45%, 13.43%, 22.01%, 59.23%, and 12.81%, respectively. These promising results provided a strong rationale for further field trials to assess the viability of ZnO-NPs as a soil amendment for safe crop production in V-contaminated environments.

**Supplementary Information:**

The online version contains supplementary material available at https://doi.org/10.1186/s12870-026-09465-y.

## Introduction

Vanadium (V) is a widely distributed trace element, accounting for approximately 0.02% of the Earth’s crust. China possesses vast reserves of vanadium minerals, notably vanadium-titanium magnetite (up to 10 billion tons) [53]. Intensive industrial activities, including iron and steel production, pharmaceuticals, and fertilizer manufacturing, have progressively increased environmental V contamination [7, 23], with soils near smelting sites containing 168-1538 mg/kg [6, 52]. In the environment, V exists in multiple oxidation states. Among these, the mobile and bioavailable vanadate [V(v)] is the most common form in aerobic soils [13]. While trace V functions as a beneficial element, promoting plant growth and metabolism (e.g., amino acids and sugars), elevated concentrations induce severe phytotoxicity [17, 42]. Excess V disrupts physiological and biochemical processes, suppresses plant biomass, alters enzyme activities, reduces nutrient uptake, inhibits chlorophyll and protein synthesis, impairs cell wall formation, and can even induce cell apoptosis [18, 20, 59]. It also stimulates overproduction of reactive oxygen species (ROS), and the resulting oxidative stress can cause chromosomal damage, metabolic dysfunction, increased cell membrane permeability, and lipid peroxidation that damages cellular structures [4, 19, 21]. As a phosphorus (P) analogue, V competitively inhibits P uptake and utilization, and disrupts the activity of key P-dependent enzymes, such as ATPase and phosphatases [22]. Moreover, V accumulation in edible tissues represents a direct risk to human health via food-chain transfer [50, 51], [59]. The persistence of V in soil poses a threat to agricultural productivity and ecosystem safety, making the development of effective remediation strategies imperative.

Engineered nanomaterials, particularly nanoparticles (NPs), offer innovative solutions for mitigating abiotic stress in crops [10, 16, 64]. Among these, zinc oxide nanoparticles (ZnO-NPs) are promising due to the essential role of zinc in plant physiology and stress tolerance [26, 48]. Studies demonstrate that ZnO-NPs alleviate stress through dual mechanisms: by modulating carbon/nitrogen metabolism and secondary metabolic pathways, and by directly mitigating heavy metal/metalloid (HM) toxicity [49]. For instance, ZnO-NPs application can reduce cadmium (Cd) uptake and oxidative stress while enhancing photosynthesis and grain yield [1, 8, 18, 31]. Similar protective effects against other metals have been observed: they enhance rice growth under chromium (Cr) stress via modulating oxidative stress-related genes [37], and improve photosynthetic apparatus functionality in maize exposed to cobalt (Co) stress [43]. Collectively, research confirms that ZnO-NPs application effectively improves biomass, phytohormone levels, photosynthetic efficiency, and free amino acid accumulation, while simultaneously reducing HM uptake in plants [2].

Grain nutritional quality, primarily determined by protein and amino acid (AAs) composition, is essential for human health [63]. Dietary protein provides amino acids necessary for tissue growth, repair, and maintenance [5]. Among these, essential amino acids (EAAs) are particularly crucial because they cannot be endogenously synthesized and must be obtained from the diet. Notably, exposure to HMs can significantly alter amino acid metabolism in plants [11]. Wheat (*Triticum aestivum* L.), a global cultivated cereal, is susceptible to HM accumulation in its edible parts. Furthermore, Zinc deficiency is widespread in wheat grown in arid and semiarid regions [38]. These intersecting concerns, including V contamination, grain nutrition, and Zn availability, highlight the urgency of developing effective strategies to mitigate V toxicity in wheat, thereby safeguarding crop production and food security. Therefore, this study aimed to evaluate the practical efficacy and physiological responses of wheat to ZnO-NPs application under V stress. While recognizing that the effects may arise from a combination of zinc nutrient and particle-specific properties, the primary goal here was to establish a proof of concept for their overall efficacy. Specifically, we sought to: (I) quantify the impact of ZnO-NPs on the V uptake, translocation, and tissue partitioning,(II) assess their effects on plant biomass, nutrient acquisition, and grain yield,and (III) elucidate the associated changes in nitrogen assimilation and amino acid profile in grains. We hypothesize that the application of ZnO-NPs will reduce V bioaccumulation and toxicity, thereby improving wheat growth and grain nutritional quality under V stress. This work paves the way for developing ZnO-NPs-based strategies to enhance crop safety and nutritional quality in V-contaminated environments.

## Materials and methods

### Experimental design and pot experiment

Topsoil (0-20 cm depth) was collected from an undisturbed agricultural field. The soil was air-dried at room temperature, passed through a 2-mm sieve, and visible plant residues were carefully removed by hand. The soil was classified as a sandy loam, with a particle size distribution of 76.2% sand, 15.5% silt, and 8.3% clay. Its basic physicochemical properties were as follows: pH (1:5 soil/water) 8.03; total organic carbon (TOC) 13.24 mg/g; total nitrogen (TN) 3.23 mg/g; available phosphorus (AP) 0.15 mg/g; available potassium (AK) 0.11 mg/g, and available Zinc (Zn) 1.32 mg/kg, respectively. The vanadium content in the soil ranged from 20 to 30 mg/kg. Sodium metavanadate (NaVO_3,_ Purity > 99%) and zinc oxide nanoparticles (ZnO-NPs) were purchased from Shanghai Macklin Inc. (China). The diameter, zeta potential, and PDI for ZnO-NPs were 30 ± 10 nm, −28.23 ± 1.5 mV, and 0.25. Before use, the morphology and elemental composition of the nanoparticles were characterized by scanning electron microscopy (SEM). Representative images are provided in Figure S1. Seeds of spring wheat (Triticum aestivum L. cv. Longchun-40) were obtained from the Gansu Academy of Agricultural Sciences.

A pot experiment was conducted using a full-factorial design with three levels of V stress (0, 100, and 200 mg V kg⁻^1^ soil, supplied as NaVO₃) and three levels of ZnO-NPs amendment (0, 100, and 200 mg kg⁻^1^ soil), resulting in nine treatment combinations (3 V levels × 3 ZnO-NPs levels). Throughout the text, treatments are codes as V(0), V(1), V(2), and ZnO(0), ZnO(1), ZnO(2). The V(0) + ZnO(0) combination served as the untreated control (CK). Plastic pots (18 cm diameter, 27 cm height) were each filled with 8.0 kg of the prepared soil, which had been uniformly amended with the corresponding concentrations of V and ZnO-NP. A basal fertilizer equivalent to 120–50-25 kg/ha of N-P_2_O_5_-K_2_O was applied using urea, diammonium phosphate, and potassium sulfate. Before soil amendment, the ZnO-NPs were dispersed in deionized water and ultrasonicated for 30 min to minimize aggregation. Wheat seeds were surface-sterilized with hydrogen peroxide, rinsed thoroughly with deionized water, and sown on 25 February 2023. The experimental design comprises five replicates per treatment. Each pot was seeded with 12 seeds, and then thinned to six uniform seedlings after germination. Plants were grown under natural conditions, with pots irrigated with tap water to maintain soil moisture at approximately 60% of field capacity.

### Plant growth conditions and physiological traits

Photosynthetic parameters and chlorophyll content were measured at 78 days after sowing. Key photosynthetic parameters, including net photosynthesis rate (*P*_*n*_), stomatal conductance (*G*_*s*_), and transpiration rate (*Tr*), were measured from the central portion of flag leaves using a portable photosynthesis instrument (Li-6400, Li-COR, USA) between 9:00 and 11:00 a.m. under stable light conditions. The relative chlorophyll content (SPAD value) of the same leaves was measured with a SPAD chlorophyll meter (SPAD-502Plus, Konica Minolta, Japan). Six measurements were taken per pot, and the average was calculated as the representative value for that pot.

Activities of nitrogen metabolism enzymes were analyzed in fresh flag leaves and developing seeds collected at the grain-filling stage. The activities of nitrate reductase (NR, EC 1.7.1.1), glutamine synthetase (GS, EC 6.3.1.2), glutamate synthase (GOGAT, EC 1.4.1.14), and glutamate dehydrogenase (GDH, EC 1.4.1.2) were determined using a commercial assay kit (Abbkine Biotechnology Co., Ltd., Wuhan, China) according to the manufacturer’s protocols. Absorbance was measured at 340 nm for NR, GOGAT, and GDH, and at 540 nm for GS, with kinetic readings taken over specified intervals (20 s for GOGAT, 5 min 20 s for GDH). Activities are expressed in units per gram fresh weight (U/g FW), where one unit (U) is defined as the consumption of 1 nmol NADH per gram FW per minute under the assay conditions.

Phytohormones were extracted and quantified as previously described with modifications [53]. Briefly, leaf samples (0.5 g) were homogenized in 5 mL of ice-cold 80% (v/v) methanol containing 1 mM butylated hydroxytoluene as an antioxidant. Indole acetic acid (IAA), cytokinin (CTK), gibberellin acid (GA), and brassinolide (BRs) were quantified using enzyme-linked immunosorbent assay (ELISA) kits, and subsequently quantified by high-performance liquid chromatography-tandem mass spectrometry (HPLC–MS/MS) according to the methods of Siciliano et al. [44, 45].

### Plant harvest, biomass, and element analysis

Plants were harvested at physiological maturity (100 days after sowing) for morphological and element analyses. Plant height and spike length were measured immediately. Subsequently, plants were separated into roots, stems, leaves, and grains. To remove surface-adsorbed metals, root samples were ultrasonically cleaned in a 20 mM Na_2_-EDTA solution for 20 min [3]. All plant tissues were oven-dried at 70℃ to constant weight (approximately 72 h), ground to a fine powder, and passed through a 100-mesh sieve for subsequent analysis.

Vanadium concentrations in plant tissues and soil were determined using inductively coupled plasma-atomic emission spectrometry (ICPS-7500, Shimadzu, Kyoto, Japan) following acid digestion. Plant powders were dry-ashed in a muffle oven at 500℃ for 5 h and then dissolved in 0.1 M HCl. Soil samples collected at the end of the experiment were homogenized and sieved (2 mm). For digestion, 0.2 g of soil was treated with 1 mL of concentrated HNO_3_ (60%, w/w) and 1 mL of HClO_4_ (60%, w/w) in a high-pressure bomb and heated at 140℃ for 6 h. All digests (plant and soil) were analyzed for V content, following established protocols with modifications [30]. A vanadium standard solution (GSB 04–1759-2004, 1 g/L) was used for calibration. The average recovery of V from the reference standard was 103% (*n* = 4). Distilled water was used as the blank in all analyses. Total zinc content in seeds was determined separately. Samples (≤ 0.2 g) were ashed at 500 °C for 6 h, dissolved in 5 mL of 1:1 HNO₃, and diluted to 50 mL. Zn concentrations were measured using an atomic absorption spectrophotometer (AA320CRT, China). Quality control with certified reference material GBW 10011, giving an average zinc recovery of 96% ± 3%.

### Calculation of V translocation from soil to plant

The translocation patterns of V in wheat plants were evaluated using the bioaccumulation factor (BAF) and biological transfer coefficient (BTC).

$$\mathrm{B}\mathrm{A}\mathrm{F} = [\mathrm{V}] \ \mathrm{p}\mathrm{l}\mathrm{a}\mathrm{n}\mathrm{t} \ \mathrm{t}\mathrm{i}\mathrm{s}\mathrm{s}\mathrm{u}\mathrm{e} (\mathrm{m}\mathrm{g}/\mathrm{k}\mathrm{g} \ \mathrm{D}\mathrm{W})/\mathrm{V} \ \mathrm{s}\mathrm{o}\mathrm{i}\mathrm{l}(\mathrm{m}\mathrm{g}/\mathrm{k}\mathrm{g})$$

where [V] plant tissue represents the V concentration in roots, shoots, glumes, or grains at maturity [25]:

The BTC of V between different plant compartments was calculated as follows

$$\mathrm{B}\mathrm{T}\mathrm{C} \ \mathrm{g}\mathrm{r}\mathrm{a}\mathrm{i}\mathrm{n}/\mathrm{g}\mathrm{l}\mathrm{u}\mathrm{m}\mathrm{e} = \mathrm{V} (\mathrm{g}\mathrm{r}\mathrm{a}\mathrm{i}\mathrm{n}) / \mathrm{V} (\mathrm{g}\mathrm{l}\mathrm{u}\mathrm{m}\mathrm{e})$$

$$\mathrm{B}\mathrm{T}\mathrm{C} \ \mathrm{g}\mathrm{l}\mathrm{u}\mathrm{m}\mathrm{e}/\mathrm{s}\mathrm{h}\mathrm{o}\mathrm{o}\mathrm{t} = \mathrm{V} (\mathrm{g}\mathrm{l}\mathrm{u}\mathrm{m}\mathrm{e}) / \mathrm{V} (\mathrm{s}\mathrm{h}\mathrm{o}\mathrm{o}\mathrm{t})$$

$$\mathrm{B}\mathrm{T}\mathrm{C} \ \mathrm{s}\mathrm{h}\mathrm{o}\mathrm{o}\mathrm{t}/\mathrm{r}\mathrm{o}\mathrm{o}\mathrm{t} = \mathrm{V} (\mathrm{s}\mathrm{h}\mathrm{o}\mathrm{o}\mathrm{t}) / \mathrm{V} (\mathrm{r}\mathrm{o}\mathrm{o}\mathrm{t})$$

$$\mathrm{B}\mathrm{T}\mathrm{C} \ \mathrm{r}\mathrm{o}\mathrm{o}\mathrm{t}/\mathrm{s}\mathrm{o}\mathrm{i}\mathrm{l} = \mathrm{V} (\mathrm{r}\mathrm{o}\mathrm{o}\mathrm{t}) / \mathrm{V} (\mathrm{s}\mathrm{o}\mathrm{i}\mathrm{l})$$

All concentrations are expressed as mg/kg dry weight [55].

### Seed nutritional quality analysis

Wheat seed samples were digested using H_2_SO_4_-H_2_O_2_ to extract nitrogen (N), phosphorus (P), and potassium (K). The N and P content in the digest was quantified using an automated continuous flow analyzer (AA3, SEAL Analytical, Germany), while K was determined via flame photometer [35]. Each sample was analyzed in duplicate.

The composition of essential and non-essential amino acids in wheat grains was analyzed by High-Performance Liquid Chromatography (HPLC) using the Pico-Tag method [5]. Briefly, homogenized seeds (200 mg) were hydrolyzed in 6 N HCl (10 mL) at 150℃ for 1 h. After hydrolysis, samples were filtered, and 1 mL was transferred to an autosampler vial for HPLC injection and quantification.

Protein nutritional quality was evaluated using the essential amino acid index (EAAI), calculated as the geometric mean of the ratio of each essential amino acid (EAA) in the sample to its corresponding values in a reference protein (egg). The EAAI calculation follows this formula:

$$\mathrm{E}\mathrm{A}\mathrm{A}\mathrm{I}=\sqrt[n]{100\frac{a}{A}\times 100\frac{b}{B}\times 100\frac{c}{C}\dots \dots 100\frac{x}{X}}$$

where *a, b, c, …,* and* x* are the concentrations of individual EAAs in the test sample; *A, B, C,* …, and *X* are the corresponding concentrations in the reference protein (egg), respectively, and *n* is the number of EAAs considered. A higher EAAI value indicates a more balanced amino acid profile and superior protein quality.

### Statistical analysis

Data are presented as mean ± SE. The main and interactive effects of V stress and ZnO-NPs amendment were evaluated by two-way analysis of variance (ANOVA). Where significant effects were detected (*P* < 0.05), post-hoc multiple comparisons were performed using Tukey’s honestly significant difference (HSD). For analysis focusing on the effect of a single factor within a subset of treatments, including nutrient elements (N, P, and K, Zn) and amino acid profiles, one-way ANOVA followed by Tukey’s HSD test was applied, with the same significance threshold (*P* < 0.05). For certain treatments, the vanadium concentration in wheat grains was below the detection limit (BDL); therefore, no measurable data points are shown. Relationships between plant physiological parameters and amino acid profiles were assessed using Pearson correlation analysis. All statistical analyses and graphical representations were obtained using GraphPad Prism software (version 10.4.1).

## Results

### Chlorophyll, photosynthesis, and phytohormones in the flag leaf

Both ZnO-NPs and V significantly influenced chlorophyll content (SPAD value), photosynthesis, and phytohormone levels in flag leaves during the seed-filling phase of wheat (Table 1). Compared with CK (no V, no ZnO-NPs), ZnO-NPs alone had no significant effect on SPAD values. While V(1) and V(2) treatments alone significantly reduced the SPAD value by 18.33% and 31.55%, respectively (*P* < 0.05), the co-application of ZnO-NPs effectively alleviated this V-induced chlorophyll degradation. For instance, the combined treatments V(1) + ZnO(1) and V(1) + ZnO(2) significantly increased the SPAD value by 12.01% and 16.50%, respectively, compared to the V(1)-only treatment (*P* < 0.05). Application of ZnO-NPs alone did not significantly alter photosynthetic efficiency (Table 1). The V(1) treatment significantly decreased the photosynthetic rate (*Pn*), stomatal conductance (*Gs*), and respiration rate (*Tr*) by 16.02%, 11.21%, and 16.95%, respectively, compared to the CK (*P* < 0.05). This inhibition was effectively alleviated by the co-application of ZnO-NPs. Notably, the combined treatment V(1) + ZnO(2) significantly enhanced *Pn*, *Gs,* and *Tr* by 10.04%, 13.80%, and 11.75%, respectively, compared to the V(1)-only treatment (*P* < 0.01).

**Table 1:** Effects of application of ZnO-NPs on chlorophyll contents, photosynthetic rate (*Pn*), transpiration rate (*Tr*), and stomatal conductance (*Gs*) at the wheat flag leaf under vanadium (V) stress

Treatment		Chlorophyll (SPAD)		*Pn* (umol CO_2_^−1^ m^−2^ s^−1^)		*Tr* (mmol m^−2^·s^−1^))		*G*_*s*_ (mol m^−2^ s^−1^)	
V(0)	ZnO(0)	39.08 ± 1.34Aa		32.12 ± 1.08Ab		2.66 ± 0.14Aa		0.19 ± 0.01Ab	
	ZnO(1)	40.30 ± 1.92Aa		32.67 ± 1.28Aab		2.52 ± 0.08Aa		0.20 ± 0.01Aa	
	ZnO(2)	39.58 ± 1.28Aa		35.05 ± 1.43Aa		2.54 ± 0.07Aa		0.22 ± 0.01Aa	
V(1)	ZnO(0)	31.91 ± 1.12Bb		27.40 ± 0.77Bb		1.93 ± 0.06Bb		0.17 ± 0.01Bb	
	ZnO(1)	35.75 ± 0.89Ba		29.94 ± 0.79Ba		2.16 ± 0.09Ba		0.19 ± 0.00Aa	
	ZnO(2)	37.18 ± 1.47Ba		29.82 ± 0.65Ba		1.65 ± 0.08Bc		0.11 ± 0.01Bc	
V(2)	ZnO(0)	26.75 ± 1.52Ca		23.51 ± 1.46Ca		1.57 ± 0.11Cc		0.11 ± 0.01Cb	
	ZnO(1)	26.18 ± 1.63Ca		23.34 ± 0.97Ca		2.17 ± 0.11Ba		0.20 ± 0.00Ba	
	ZnO(2)	27.31 ± 1.96Ca		24.46 ± 1.25Ca		1.96 ± 0.06Cb		0.11 ± 0.00Bb	
Factor		*F*	*P*	*F*	*P*	*F*	*P*	*F*	*P*
V		136.5	***	122.0	***	373.3	***	806.3	***
ZnO		2.025	*	3.884	*	31.30	**	113.5	***
V*ZnO		24.46	***	14.17	***	46.84	***	308.5	***

Data are presented as means ± SE (*n* = 5). Different uppercase letters indicate significant differences among ZnO-NPs addition levels at the same V stress level, and different lowercase letters indicate significant differences among V stress levels at the same ZnO-NPs level (two-way ANOVA, Tukey's HSD test, ***, *P* < 0.001; **, *P* < 0.01; *, *P* < 0.05). ZnO(0), ZnO(1), and ZnO(2) correspond to soil ZnO-NPs concentrations of 0, 100, and 200 mg/kg, respectively; V (0), V(1) and V(2) correspond to soil V concentrations of 0, 100, and 200 mg V kg^−1^, respectively

**Fig. 1:**
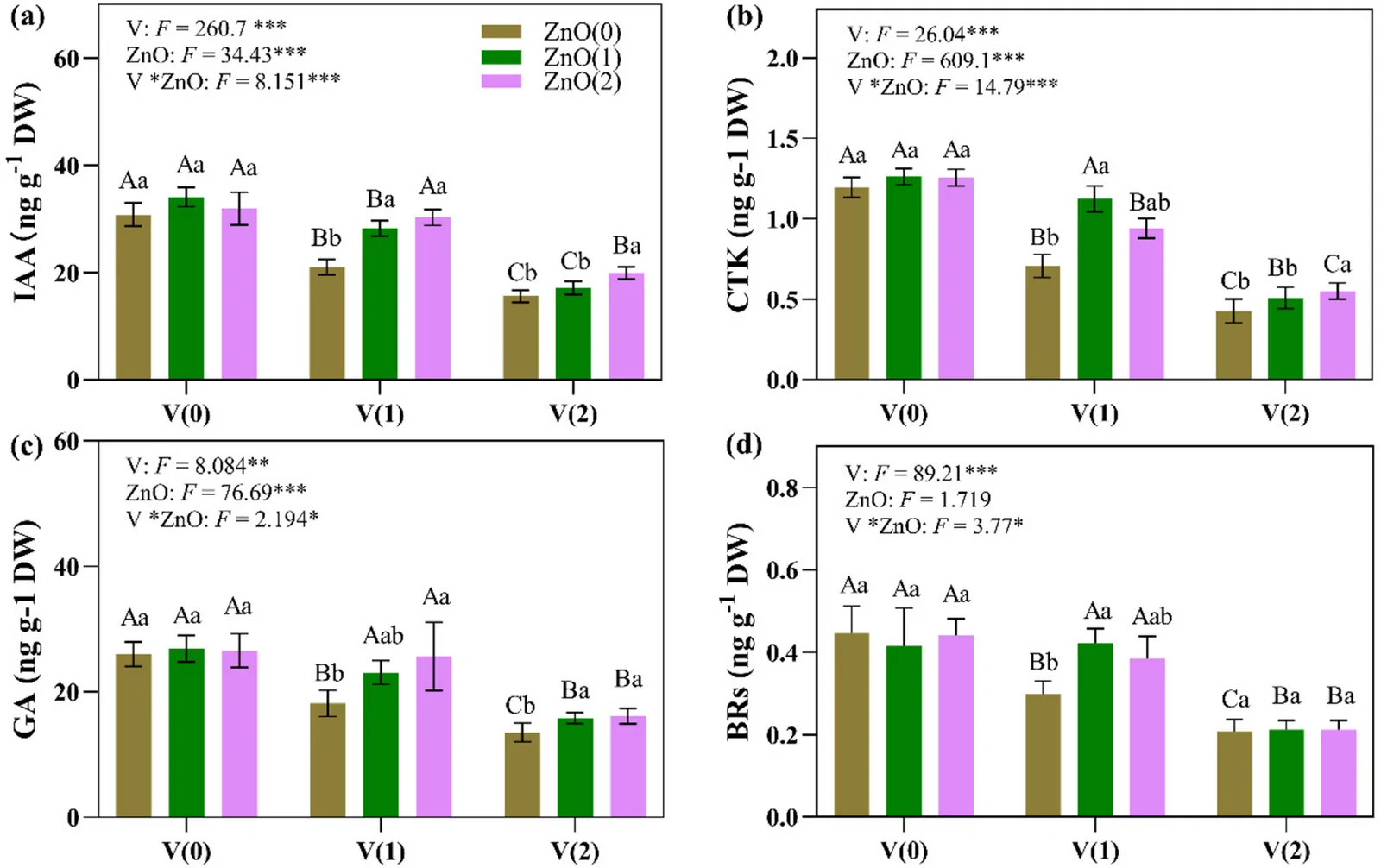
Effects of ZnO-NPs on the phytohormone contents in wheat flag leaf under vanadium (V) stress. **a** Indole acetic acid (IAA); **b** Cytokinin (CTK); **c** Gibberellic acid (GA), and **d** Brassinolide (BRs) Data are means ± standard error (SE, n = 5). Different uppercase letters indicate significant differences among ZnO-NPs levels within the same V level, different lowercase letters indicate significant differences among V levels within the same ZnO-NPs level (two-way ANOVA, Tukey's HSD test, ***P < 0.001, **P < 0.01, *P < 0.05). ZnO(0), ZnO(1), and ZnO(2) indicate the addition of 0, 100, and 200 mg kg^-1^ ZnO-NPs, respectively; V (0), V(1), and V(2) indicate the addition of 0, 100, and 200 mg V kg^-1^ V, respectively

The phytohormone contents (IAA, CTK, GA, and BRs) in the flag leaf were significantly influenced by both V and ZnO-NPs (Fig. 1, *P* < 0.01). Application of ZnO-NPs alone caused no significant reduction in these phytohormones (Fig. 1a-d). Compared to CK, the V(1) treatment significantly decreased IAA, CTK, GA, and BRs by 31.79%, 40.70%, 30.08%, and 33.18%, respectively (*P* < 0.01). These declines were effectively alleviated by co-application with ZnO-NPs. The combined treatment V(1) + ZnO(2) increased IAA and GA levels by 44.35% and 41.11%, respectively (*P* < 0.01). The V(1) + ZnO(1) treatment significantly increased CTK and BRs by 58.75% and 41.61%, respectively (*P* < 0.01). Moreover, no significant improvements in CTK, GA, and BRs were observed when ZnO-NPs were applied under V(2) stress.

### Activities of enzymes related to nitrogen metabolism at the filling phase

**Fig. 2:**
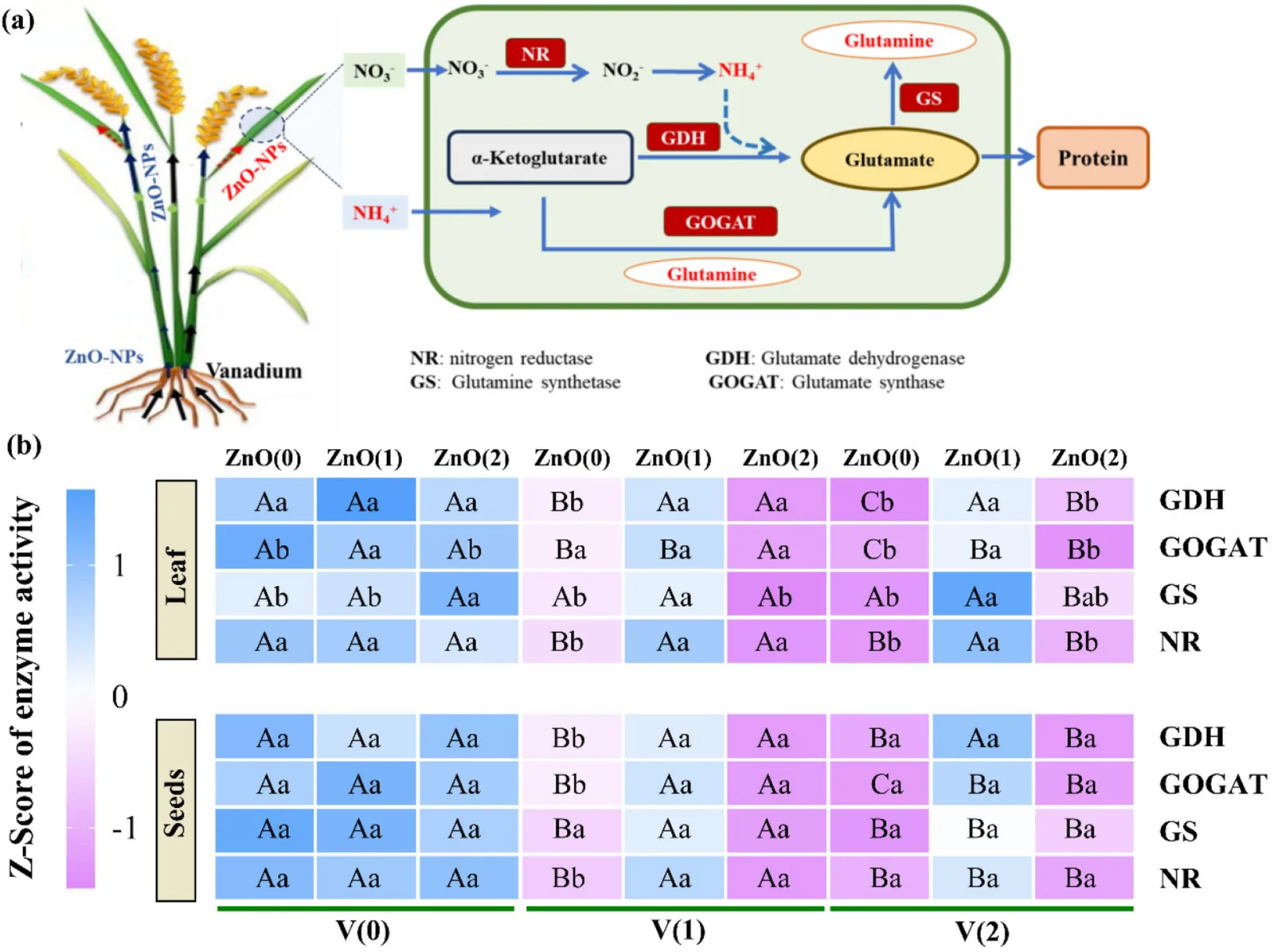
Effects of ZnO-NPs on nitrogen metabolism enzyme activities in wheat flag leaf and seed under V stress at the filling phase. **a** Schematic diagram illustrating the key enzymes in the nitrogen assimilation pathway. **b** Heat map of enzyme activities across treatments; color intensity reflects normalized enzyme activity. Significant differences were determined by two-way ANOVA followed by Tukey's HSD test (P < 0.05. Different uppercase letters indicate significant differences among ZnO-NPs levels within the same V level, and different lowercase letters indicate significant differences among V levels within the same ZnO-NPs level. ZnO(0), ZnO(1), and ZnO(2) indicate the addition of 0, 100, and 200 mg kg^-1^ ZnO-NPs, respectively; V (0), V(1), and V(2) indicate the addition of 0, 100, and 200 mg V kg^-1^ V, respectively

The activities of key nitrogen metabolism enzymes, including nitrate reductase (NR), glutamine synthetase (GS), glutamate synthase (GOGAT), and glutamate dehydrogenase (GDH), in the flag leaves and seeds were influenced by V and ZnO-NPs. Under V(0) conditions, ZnO-NPs applied induced no significant alterations in enzyme activities in either tissue compared to CK. Vanadium stress inhibited these activities, and co-application of ZnO-NPs effectively alleviated the inhibition under moderate V(1) but not under V(2) stress (Fig. 2). In flag leaves, the V(1) treatment significantly reduced the activities of NR, GS, GOGAT, and GDH by 25.96%, 8.76%, 38.10%, and 31.77%, respectively, compared to the CK (*P* < 0.01). The combined treatment V(1) + ZnO(2) significantly increased the activities of NR, GS, GOGAT, and GDH by 37.62%, 27.62%, 15.78%, and 21.08%, respectively, compared to the V(1)-only treatment (Fig. 2, *P* < 0.01), restoring them to levels comparable with the CK groups. A parallel response was observed in developing seeds at the filling stage. Here, the V(1) treatment significantly reduced NR, GS, GOGAT, and GDH activities by 30.82%, 42.84%, 31.27%, and 34.62%, respectively, compared to CK (Fig. 2, *P* < 0.01). The combined treatment V(1) + ZnO(2) significantly increased the NR, GS, GOGAT, and GDH activities by 18.89%, 44.49%, 40.42%, and 46.40%, respectively, compared to the V(1)-only treatment (Fig. 2, *P* < 0.01).

### Agronomic traits of wheat at a mature stage

**Fig. 3:**
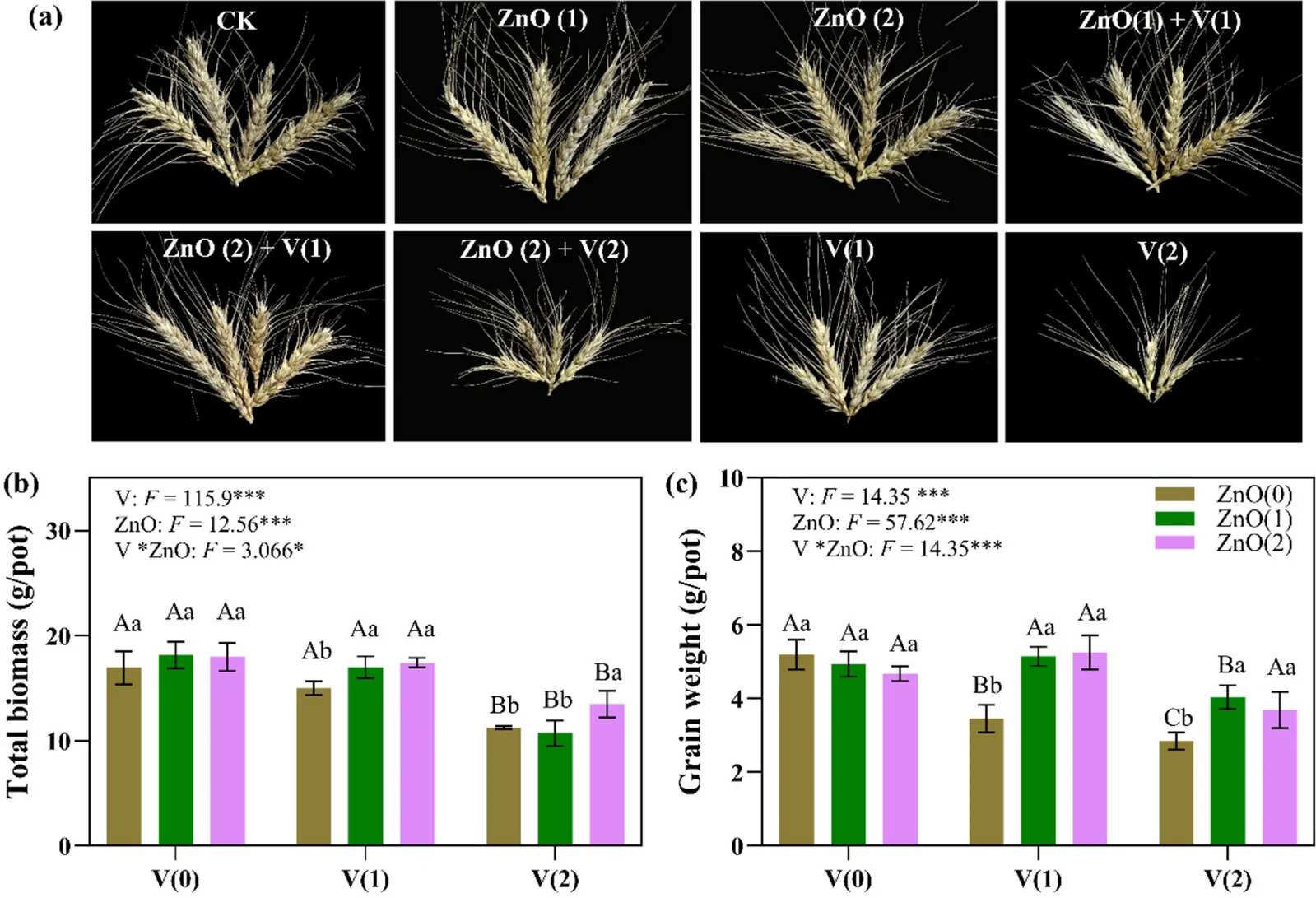
Effects of ZnO-NPs on wheat growth at maturity under vanadium (V) stress. **a** Representative ear phenotype (four ears per treatment shown), **b** Total biomass per pot, **c** Grain yields per pot. Data are means ± standard error (SE, n = 5). Different uppercase letters indicate significant differences among ZnO-NPs levels under the same V level, and different lowercase letters indicate significant differences among V levels under the same ZnO-NPs level (two-way ANOVA, Tukey's HSD test, ***P < 0.001, **P < 0.01, *P < 0.05). ZnO(0), ZnO(1), and ZnO(2) indicate the addition of 0, 100, and 200 mg kg-1 ZnO-NPs, respectively; V (0), V(1), and V(2) indicate the addition of 0, 100, and 200 mg V kg-1 V, respectively

Both ZnO-NPs and V significantly influence key agronomic traits at maturity (Table 2 and Fig. 3). In the absence of V, ZnO-NPs applied had no significant effect on growth or yield parameters. Vanadium exposure significantly inhibits both plant growth and yield formation. The V(1) treatment significantly reduced the plant height, above-ground biomass, below-ground biomass, and total biomass by 13.25%, 11.17%, 33.23%, and 12.58%, respectively, compared to the CK (Table 2). Concurrently, the V (1) treatment significantly decreased spike length, kernel number per ear, and grain weight per pot by 29.98%, 16.45%, and 31.94%, respectively, compared to the CK (Table 2, *P* < 0.05). Co-application of ZnO-NPs effectively alleviates this V-induced inhibition. For example, the combined treatment V(1) + ZnO(1) significantly increased aboveground biomass, below-ground biomass, and total biomass by 12.72%, 30.54%, and 13.60%, respectively, compared to the V(1)-only treatment (Fig. 3, *P* < 0.05). In parallel, the same combination significantly increased spike length, kernel number per ear, and grain weight per pot by 39.72%, 23.96%, and 44.95%, respectively, compared to the V(1)-only treatment (*P* < 0.01).

**Table 2:** Addition of ZnO-NPs on wheat growth at the mature stage under vanadium (V) stress

Treatment		Plant height(cm)		Spike length (cm)		Number of ears		Aboveground biomass(g)		Underground biomass(g)	
V(0)	ZnO(0)	61.49 ± 2.89Aa		8.45 ± 0.56Aa		22.17 ± 1.53Aa		16.09 ± 1.37Aa		1.10 ± 0.10Aa	
	ZnO(1)	63.44 ± 3.24Aa		8.32 ± 0.48Aa		24.00 ± 1.59Aa		16.77 ± 1.25Aa		1.04 ± 0.06Aa	
	ZnO(2)	61.37 ± 3.04Aa		8.50 ± 0.40Aa		21.81 ± 0.54Aa		16.96 ± 1.11Aa		1.02 ± 0.05Aa	
V(1)	ZnO(0)	53.44 ± 2.95Bb		5.92 ± 0.42Bb		18.52 ± 0.99Bb		14.29 ± 0.66Ab		0.74 ± 0.05Bb	
	ZnO(1)	52.45 ± 3.31Bb		8.27 ± 0.54Aa		22.96 ± 1.04Aa		16.11 ± 0.83Aa		0.96 ± 0.05Aa	
	ZnO(2)	58.38 ± 2.76Aa		8.12 ± 0.47Aa		22.81 ± 0.77Aa		16.46 ± 0.37Ba		0.91 ± 0.03Ba	
V(2)	ZnO(0)	32.44 ± 3.52Cc		3.73 ± 0.37Cc		12.27 ± 1.10Cb		10.77 ± 0.32Bb		0.63 ± 0.06Bb	
	ZnO(1)	44.19 ± 2.79Cb		5.45 ± 0.35Bb		18.31 ± 1.13Ba		10.05 ± 0.99Bb		0.78 ± 0.03Ba	
	ZnO(2)	51.68 ± 3.15Ba		6.07 ± 0.32Ba		18.31 ± 2.22Aa		13.06 ± 1.07Ba		0.44 ± 0.04Cc	
Factors		*F*	*P*	*F*	*P*	*F*	*P*	*F*	*P*	*F*	*P*
V		191.2	***	187.4	***	98.51	***	128.3	***	257.7	***
ZnO		38.22	***	89.54	***	32.87	***	15.40	***	20.56	***
V *ZnO		19.08	***	12.33	***	8.864	***	4.323	***	26.05	***

Data are presented as mean ± standard error (SE, n = 5). Different uppercase letters indicate significant differences among ZnO-NPs levels within the same V level, and different lowercase letters indicate significant differences among V levels within the same ZnO-NPs level (two-way ANOVA, Tukey's HSD test, ***P < 0.001, **P < 0.01, *P < 0.05. ZnO(0), ZnO(1), and ZnO(2) correspond to soil ZnO-NPs concentrations of 0, 100, and 200 mg/kg, respectively; V (0), V(1), and V(2) correspond to soil V concentrations of 0, 100, and 200 mg V kg^−1^, respectively

### Vanadium accumulation and translocation in wheat at the mature stage

Vanadium was detected in all examined wheat tissues (grains, glumes, stems, leaves, and roots) across all treatments (Figure S3). Tissue V concentrations consistently followed the order: roots > stems > leaves > glumes > grains. Both V and ZnO-NPs significantly influenced V accumulation in these tissues, with ZnO-NPs reducing and V addition increasing V concentration in these tissues (Figure S3, *P* < 0.05). The application of ZnO-NPs effectively reduced V accumulation across all examined tissues. The combined treatment V(1) + ZnO(1) significantly reduced V concentration by 37.24% in grain, 32.67% in leaves, 37.83% in stems, and 27.02% in roots, compared to the V(1)-only treatment (Figure S3a, d, e; *P* < 0.01). Similarly, the treatment V(1) + ZnO(2) significantly reduced V concentration by 31.38% in grain, 40.85% in stems, and 49.98% in roots (Figure S3a, d, e; *P* < 0.01).

**Fig. 4:**
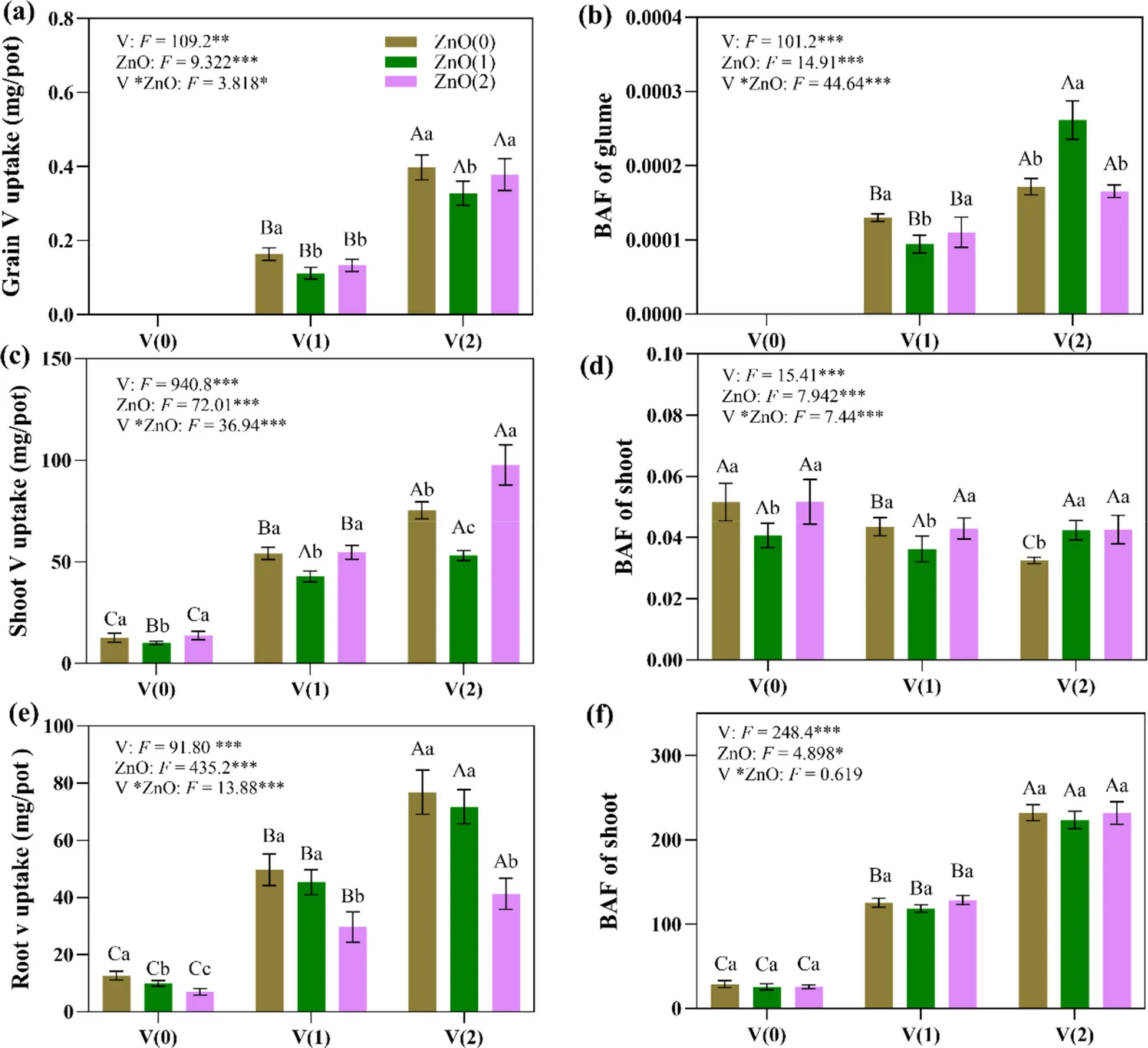
Effects of ZnO-NPs on Vanadium (V) uptake and bioaccumulation in wheat. Total V uptake in grain (**a**), shoot (**c**), and root (**e**); Bioaccumulation factor (BAF) for grain (**b**), shoot (**d**), and root (**f**). Data are means ± standard error (SE, n = 5). Different uppercase letters indicate significant differences among ZnO-NPs levels under the same V level, and different lowercase letters indicate significant differences among V levels under the same ZnO-NPs level (two-way ANOVA, Tukey's HSD test, ***P < 0.001, **P < 0.01, *P < 0.05). ZnO(0), ZnO(1), and ZnO(2) indicate the addition of 0, 100, and 200 mg kg-1 ZnO-NPs respectively; V (0), V(1) and V(2) indicate the addition of 0, 100, and 200 mg V kg-1 V, respectively

The uptake and the bioaccumulation of V in grain, shoot, and root tissues were significantly influenced by V and ZnO-NPs (Fig. 4). While V stress increased the bioaccumulation factor (BAF), co-application of ZnO-NPs significantly reduced both V uptake and BAF. Compared to V(1)-only treatment, the combination V(1) + ZnO(1) significantly decreased total V uptake by 32.46% in grain and 21.26% in shoot, respectively (Fig. 4a and c, *P* < 0.01), and reduced the BAF by 28.34% in grain and 16.73% in shoot, respectively (Fig. 4b and d, *P* < 0.01). Similarly, the combination V(1) + ZnO(2) significantly decreased the total root V uptake by 40.76% and root BAF by 42.44%, respectively (Fig. 4e and f, *P* < 0.01).

**Fig. 5:**
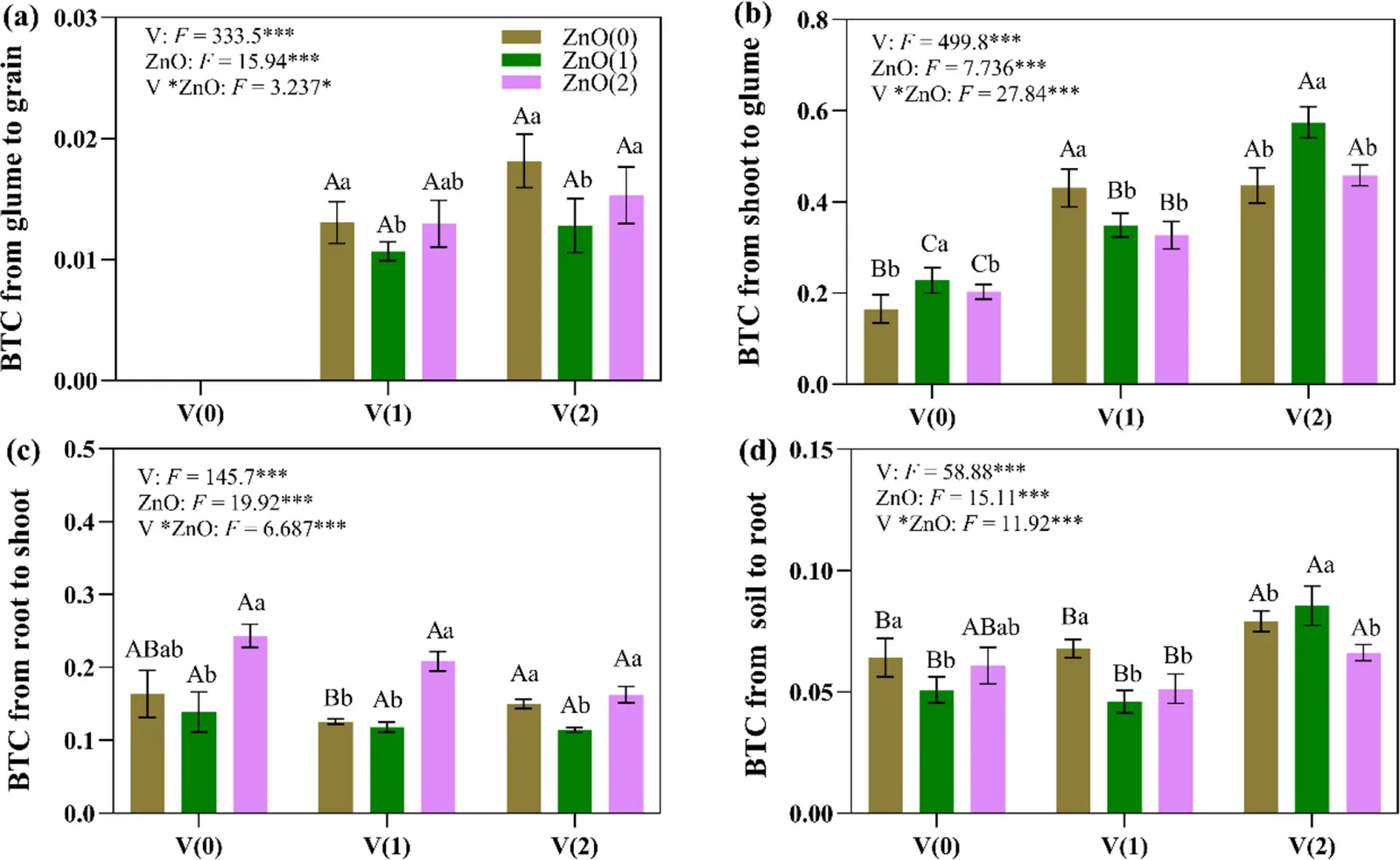
Effects of ZnO-NPs on the biological transfer coefficient (BTC) of vanadium in wheat at the mature stage. **a** BTC from glume to grain; **b** BTC from shoot to glume; **c** BTC from root to shoot; **d** BTC from soil to root. Data are presented as mean ± standard error (SE, n = 5). Different uppercase letters indicate significant differences among ZnO-NPs levels under the same V level, and different lowercase letters indicate significant differences among V levels under the same ZnO-NPs level (two-way ANOVA, Tukey's HSD test, ***P < 0.001, **P < 0.01, *P < 0.05). ZnO(0), ZnO(1), and ZnO(2) indicate the addition of 0, 100, and 200 mg kg-1 ZnO-NPs, respectively; V (0), V(1), and V(2) indicate the addition of 0, 100, and 200 mg V kg-1 V, respectively

The biological transfer coefficient (BTC) of V was also significantly influenced by both V and ZnO-NPs. The application of ZnO-NPs significantly reduced the BTC of V from glume to grain, shoots to glumes, and soil to root (Fig. 5). Compared to the V(1)-only treatment, the combination V(1) + ZnO(1) significantly decreased the BTC of V from glume to grain by 18.24% (Fig. 5a, *P* < 0.01). The combinations V(1) + ZnO(1) and V(1) + ZnO(2) significantly decreased the BTC of V from shoot to glume by 19.61% and 24.73%, respectively (Fig. 5b, *P* < 0.01). Additionally, the combinations V(1) + ZnO(1) and V(1) + ZnO(2) significantly reduced the BTC of V from soil to root by 31.49% and 22.57%, respectively (Fig. 5d, *P* < 0.01).

### Macro-nutrients and amino acid profile of wheat seeds at a mature stage

**Fig. 6:**
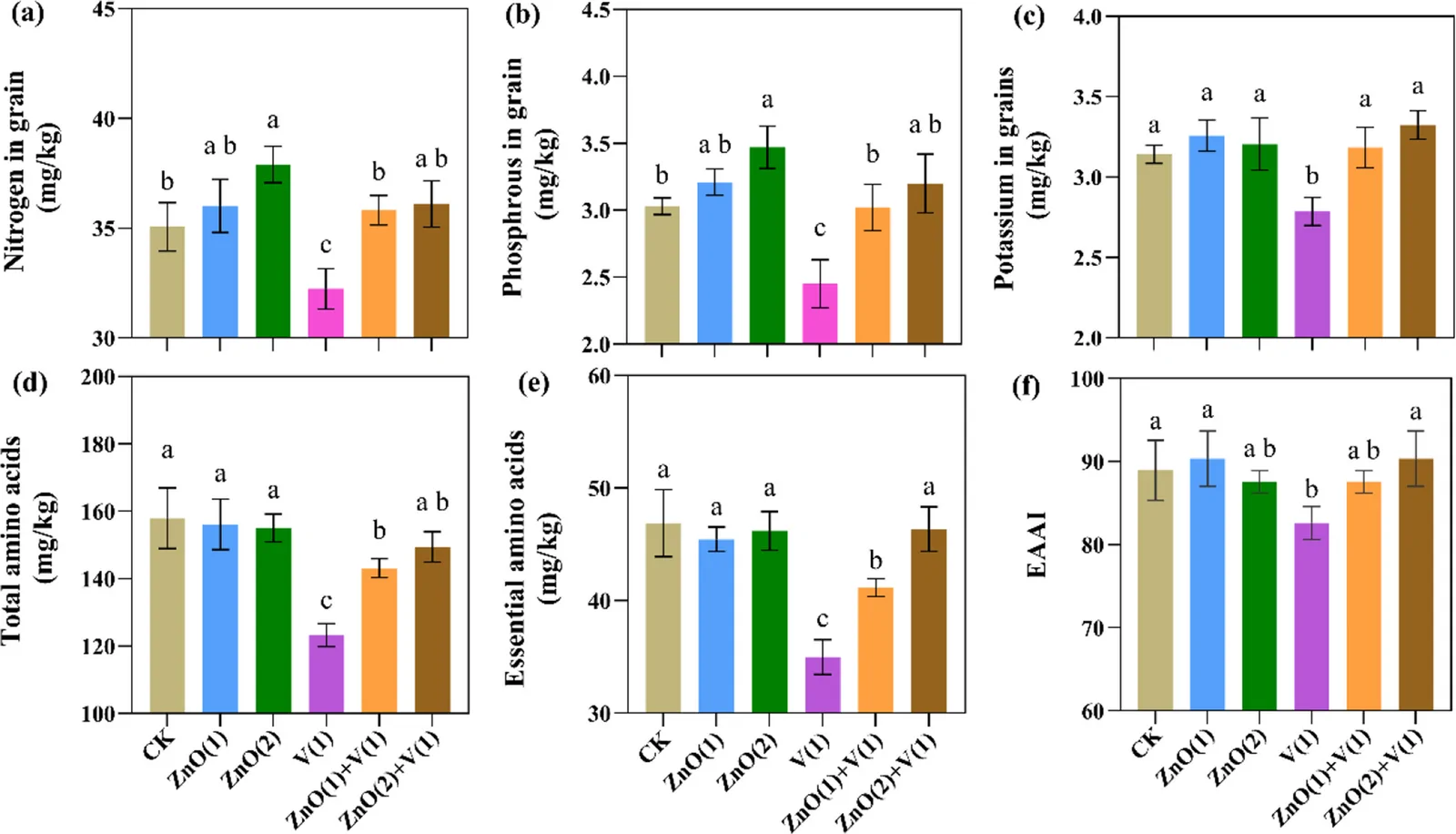
Effects of ZnO-NPs on nutrient and amino acid contents in wheat grains. **a** total nitrogen, **b** total phosphorus, **c** total potassium, **d** total amino acids, **e** essential amino acids, **f** essential amino acids index (EAAI). Data are means ± standard error (SE, n = 5). Different lowercase letters in each column indicate significant differences across treatments based on one-way ANOVA followed by Tukey’s HSD test (*P* < 0.05)

To assess the nutritional impacts, we quantified macronutrients (N, P, K) and amino acid profiles in wheat seeds from plants treated with ZnO-NPs and V(1) treatments (Fig. 6). Vanadium stress significantly reduced seed nutrient content. The V(1) treatment significantly decreased N, P, and K concentrations by 8.63%, 19.12%, and 12.73%, respectively, compared to the CK (Fig. 6a-c, *P* < 0.05). Co-application of ZnO-NPs effectively alleviated this inhibitory effect. The combined treatment V(1) + ZnO(1) significantly increased the concentrations of N, P, and K by 11.64%, 22.01%, and 13.98%, respectively, compared to the V(1) treatment (Fig. 6a-c, *P* < 0.05). Seed Zn content was significantly reduced by vanadium stress, but recovered with ZnO-NPs application. The V(1) treatment decreased Zn content by 37.90%, compared to the CK. The combinations V(1) + ZnO(1) and V(1) + ZnO(2) increased the Zn content significantly by 75.71% and 98.13%, respectively, relative to the V(1) treatment (Figure S4, *P* < 0.05).

Analysis of 17 amino acids in the grains revealed that V(1) stress also significantly impaired grain protein quality, reducing the total amino acid (TAA), essential amino acids (EAA), and the essential amino acid index (EAAI) by 21.94%, 25.39%, and 7.12%, respectively, compared to CK (Fig. 6d-f, *P* < 0.05). This inhibition was effectively mitigated by co-application with ZnO-NPs. In comparison, the combination V(1) + ZnO(2) showed a significant increase of 21.25%, 32.06%, and 9.36% in TAA, EAA, and EAAI, respectively, compared to the V(1) treatment.

**Fig. 7:**
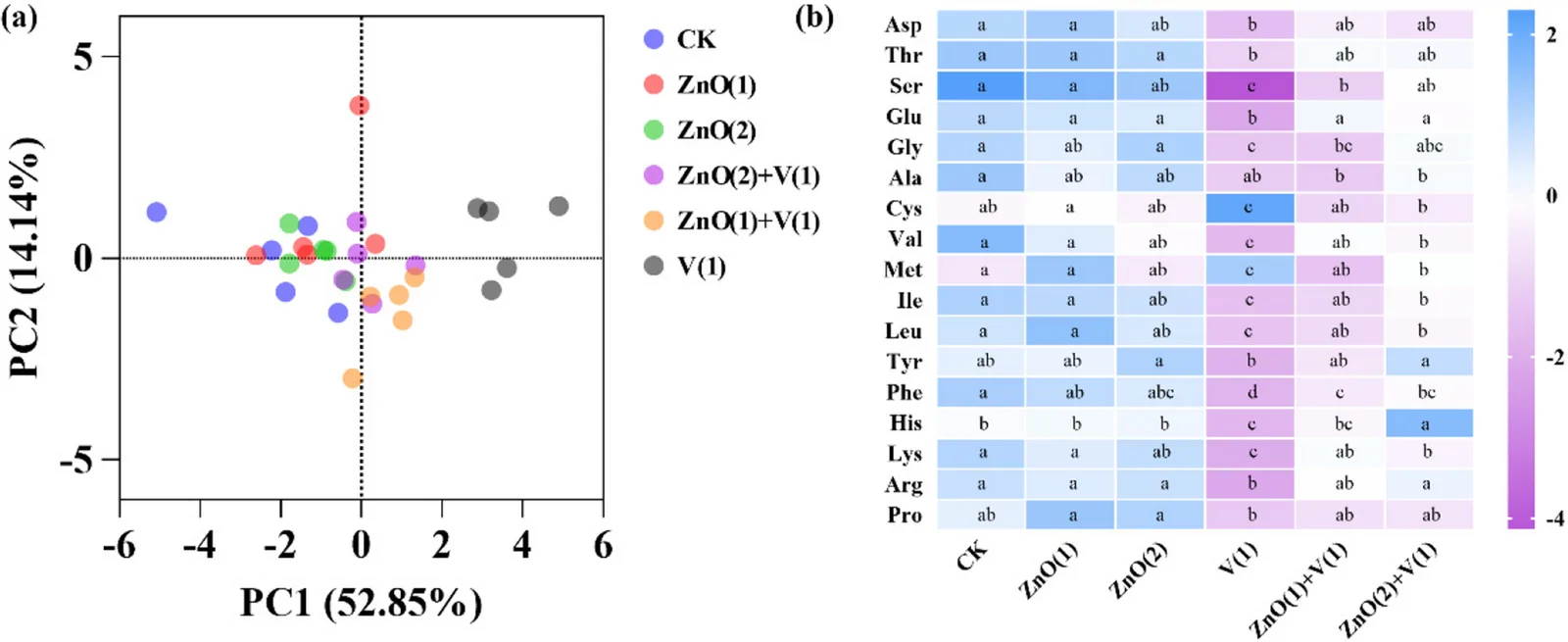
Effects of ZnO-NPs on amino acid composition in wheat grains under V stress. **a** Principal component analysis of amino acid profiles. **b** Heatmap showing normalized amino acid content across treatments, with color intensity representing the relative abundance. Different lowercase letters in the heatmap indicate significant differences among treatments based on one-way ANOVA followed by Tukey’s HSD-test (*P* < 0.05)

Principal component analysis (PCA) of the amino acid profiles revealed distinct separation among treatments, with the first two principal axes explaining 66.99% of the total variation (Fig. 7a). The V(1) treatment led to a significantly decline in six essential amino acids: valine (Val), isoleucine (Ile), leucine (Leu), phenylalanine (Phe), histidine (His), and lysine (Lys) decreased by 22.36%, 19.04%, 18.92%, 28.01%, 71.52% and 18.92%, respectively, compared to the CK (Fig. 7b). Co-application of ZnO-NPs significantly increased the content of six essential amino acids. The combination V(1) + ZnO(2) significantly increased these amino acids by 12.55% (Val), 12.45% (Ile), 13.43% (Leu), 22.01% (Phe), 59.23% (His), and 12.81% (Lys), respectively, compared to the V(1) treatment (Fig. 7b, *P* < 0.05). Furthermore, correlation analysis revealed that the contents of Val, Ile, Leu, Phe, His, and Lys were significantly correlated with most plant variables, except for NR and GS activities in flag leaves (Figure S5, *P* < 0.05).

## Discussion

### Effects of ZnO-NPs on the growth of wheat under V stress

Elevated soil vanadium (V) poses a significant threat to crop productivity and food security [21, 23, 57]. Minimizing V uptake and accumulation is therefore critical for alleviating its phytotoxicity. Vanadium enters roots primarily through transporters for essential nutrients, with its structural analogy to phosphate (P) leading to direct competitive inhibition at the uptake site [4, 65]. Our study reveals that ZnO-NPs application significantly enhanced P accumulation in wheat (Fig. 6b). The ZnO-NP-induced P enrichment intensified the P–V competition at the root interface, thereby reducing V influx and alleviating its primary toxicity.

Consistent with previous reports, V exposure severely inhibited wheat growth and yield. Our work demonstrates that ZnO-NPs can effectively counteract this inhibition. The restorative effect of ZnO-NPs is attributed to the physiological roles of zinc, an essential element that regulates key starch synthesis enzymes, thereby sustaining carbon flow toward starch biosynthesis during the grain-filling stage [12, 24]. Furthermore, by improving nitrogen use efficiency, Zn indirectly supports the biosynthesis of starch and proteins, collectively contributing to increased final grain weight [57], as observed in our study (Table 2). This regulatory role is crucial under V stress, which disrupts normal assimilate partitioning. In line with findings that lower ZnO-NPs levels promote plant growth under metal stress [39] our results confirm that ZnO-NPs application under V stress significantly increases biomass and grain yield (Table 2 and Fig. 3). Given that flag leaf is a key determinant of final yield due to its role in light interception and assimilate supply, we focused on it to elucidate the physiological basis of the observed recovery [32, 33]. Our results show that ZnO-NPs significantly improved photosynthetic efficiency and chlorophyll content in the flag leaves under V stress (Table 1). Enhanced chlorophyll levels support chloroplast structure and function, which govern photosynthetic efficiency and overall plant productivity [9, 41]. In this study, the enhancement in photosynthetic rate, stomatal conductance, and respiration rate in the V(1) + ZnO(2) treatment (Table 1) provides a direct physiological explanation for the concurrent increases in aboveground, underground, and total biomass (Table 2 and Fig. 3b). Thus, the restorative effect of ZnO-NPs operates not only by limiting V entry at the root but also by actively repairing photosynthetic source strength in the canopy Table 3.

**Table 3:** Summary of two-way ANOVA for nitrogen metabolism enzyme activities in the flag leaf and seeds at the filling stage. Significance levels (Tukey's HSD test) are indicated as ***P < 0.001, **P < 0.01, *P < 0.05

Factor	NR_leaf		GS_leaf		GOGAT_leaf		GDH_leaf	
	F	P	F	P	F	P	F	P
V	13.77	**	29.10	***	57.32	***	10.73	***
ZnO	55.78	**	14.67	**	40.28	***	4.751	*
V*ZnO	20.65	**	7.471	**	3.169	ns	87.08	***
	NR_seed		GS_seed		GOGAT_seed		GDH_seed	
	F	P	F	P	F	P	F	P
V	74.32	***	139.7	***	222.4	***	155.5	***
ZnO	2.494	*	0.837	*	10.48	*	6.251	*
V*ZnO	4.621	*	7.150	**	4.578	*	23.26	***

### Effects of ZnO-NPs on nitrogen assimilation in leaves and seeds under V stress

Given the functional coupling between nitrogen assimilation and photosynthesis, we analyzed key nitrogen assimilation enzymes, including NR, GS, and GOGAT, in flag leaves under V stress and ZnO-NPs application. Our results show that ZnO-NPs significantly enhanced the activities of GS, GOGAT, and GDH under V stress (Fig. 2), indicating a stimulated nitrogen assimilatory pathway that helps alleviate V-induced inhibition. Zinc application enhances nitrogen metabolism primarily by upregulating GS activity, thereby promoting the assimilation of ammonium nitrogen, reducing ammonia toxicity, and stimulating the synthesis of essential amino acids such as glutamate. The increased activity of GS under ZnO-NPs treatment enhanced the conversion of NH_4_^+^ and 2-oxoglutarate to glutamate, directly linking the improved nitrogen assimilation to the established role of Zinc in stimulating this central metabolic pathway [50, 51]. The increase in GDH activity, which catalyzes the reversible synthesis of glutamate from 2-hydroxoglutarate with NH_4_^+^, thus maintains glutamate availability under stress [29]. The significant boost in these enzyme activities, particularly under the combined treatment V(1) + ZnO(2), suggests that ZnO-NPs actively promote nitrogen assimilation in flag leaves beyond merely counteracting V toxicity (Fig. 2). The resulting accumulation of glutamine and glutamate serves as critical nitrogen donors for the synthesis of chlorophyll precursor, amino acids, and other key intermediates in the tricarboxylic acid cycle [27].

The accumulation of glutamine and glutamate in leaves has direct implications for nitrogen allocation to developing grains. In cereals, glutamine acts as the primary nitrogen transport molecule from vegetative organs (e.g., leaves, stems) to reproductive sinks [54, 56]. Our results show that the combined treatment ZnO(1) + V(1) significantly increased total seed nitrogen by 11.64% compared to the V(1) treatment (Fig. 6a), indicating enhanced nitrogen translocation. Within the seed, the imported nitrogen was actively reassimilated, as evidenced by significantly elevated activities of GS, GOGAT, and GDH in seeds (Fig. 2), reflecting an enhanced capacity for nitrogen reassimilation and interconversion. Glutamate, derived from glutamine, acts as a key precursor that fuels branched biosynthetic networks responsible for nitrogen redistribution and amino acid diversification [6]. Consequently, total seed amino acid content was significantly higher under the combined treatment V(1) + ZnO(2) (Fig. 6d). This increase can be attributed to elevated glutamine and glutamate levels resulting from enhanced GS-GOGAT-GDH cycle activity in both leaves and seeds induced by ZnO-NPs. In summary, ZnO-NPs mitigate V toxicity by enhancing nitrogen assimilation in two key compartments: they power primary metabolism in flag leaves [27] while optimizing storage in developing grains. This dual enhancement collectively drives improvements in photosynthesis, yield, and overall plant resilience under V stress.

### Effects of ZnO-NPs on grain nutritional quality under V stress

Amino acids in grains constitute vital nutritional components for humans and livestock. Malnutrition often results from a deficiency in essential amino acids (EAAs), which cannot be synthesized de *novo* in humans [58]. In this study, V stress significantly reduced the contents of several EAAs (Val, Ile, Leu, Phe, His, and Lys) in wheat seeds, while ZnO-NPs application restored the EAA levels and improved grain quality, as reflected by the Essential Amino Acid Index (EAAI) under V stress (Figs. 6f and 7b). Phytohormones play a crucial regulatory role in determining grain quality, including the biosynthesis of amino acids in cereals [15, 62]. Our results indicate that the recovery of EAA levels by ZnO-NPs is closely associated with the rebalancing of these hormonal pathways (Figure S5). Among these, brassinosteroids (BRs) enhance amino acid biosynthesis, potentially by upregulating key enzymes such as aspartate kinase (AK), which initiates the biosynthesis of aspartate-derived amino acids, such as lysine (Lys) and isoleucine (Ile). For example, spraying of 24-EBL (an active BRs) during the grain-filling stage in rice significantly elevates AK activity and increases total amino acids in rice [54, 56]. Furthermore, improved nitrogen management has been shown to elevate endogenous BR levels in filling grains, concomitant with increased contents of essential amino acids (EAA) and Lys [60, 61]. In line with these findings, our data revealed significant positive correlations between Lys and Ile in wheat seeds and the endogenous BRs (Spearman’s *ρ* = 0.63 and 0.40, *P* < 0.05, Figure S5).

Gibberellic acid (GA) and cytokinin (CTK) are also important mediators of amino acid biosynthesis [34, 36]. Exogenous GA application has been shown to improve the amino acid profiles, increasing Val, Leu, and His in crops [46, 47]. Furthermore, under high salinity stress, CTK can enhance the accumulation of Leu, Lys, and Val in Bean [14]. Our results showed that Val, Leu, and His significantly positively correlated with the GA (Spearman’s *ρ* = 0.70, 0.60, and 0.47, *P* < 0.05, Figure S5). In parallel, CTK levels correlated positively with Lys, Leu, and Val (Spearman’s *ρ* = 0.62, 0.77, and 0.54, *P* < 0.05, Figure S5). Collectively, these findings suggest that ZnO-NPs improve the EAA content and nutritional quality of wheat seeds under V stress by modulating endogenous phytohormone pathways, particularly those involving BRs, GA, and CTK, which function to enhance the production and accumulation of essential amino acids.

### Limitations and further perspectives

This study demonstrates that ZnO-NPs can effectively alleviate V stress in wheat by enhancing nitrogen assimilation and grain nutritional quality. However, a fundamental question remains: the specific contributions of Zn^2^⁺ nutrition, particle-size effects, and distinct nano-specific properties to these improvements require clarification. To disentangle these mechanisms, further studies should employ systematic comparisons, such as parallel treatments with ionic Zn salts, bulk ZnO, and comparably sized inert nanoparticles. Such insights are essential for rationally nano-enabled strategies. Furthermore, field-scale validation is the next step to assess the agronomic feasibility, long-term stability, and environmental behavior of ZnO-NPs under real-field conditions. Addressing these questions will pave the way for translating nano-enabled stress mitigation from a promising concept into a targeted and sustainable agricultural practice.

## Conclusion

This study demonstrates that zinc oxide nanoparticles (ZnO-NPs) alleviate vanadium (V) toxicity in wheat by reducing its uptake and translocation to grains. Concurrently restore photosynthetic capacity, nitrogen assimilation, and phytohormone balance in flag leaves. These physiological improvements activated key biosynthetic pathways, recovering biomass and yield while significantly enhancing grain nutritional quality. The increase in essential amino acids (e.g., valine, isoleucine, and histidine) is directly linked to this phytohormonal rebalancing, indicating that ZnO-NPs reprogram seed composition by modulating internal signaling and metabolism. Overall, the ZnO-NPs not only reduce contaminant accumulation and actively reprogram seed nutritive value, supporting their potential as an effective intervention for crop production in V-contaminated environments. Therefore, the mechanistic insights elucidated here pave the way for the field-scale application of this promising nanotechnology in sustainable agriculture.

## Supplementary Information

Supplementary Material 1

## Data Availability

The authors declare that the data supporting the findings of this study are available within the paper. The datasets used and analyzed during the current study could be available from the corresponding author upon reasonable request.
