# Supplementary material for: Zinc nanoparticles alleviate vanadium stress in wheat (Triticum aestivum L.): impacts on vanadium accumulation, nitrogen assimilation, and amino acid profile
Source: BMC Plant Biol. 2026 Jul 13;26:1714. doi: 10.1186/s12870-026-09465-y (PMC13647550; doi:10.1186/s12870-026-09465-y)
Supplement: Supplementary file 1 [file 12870_2026_9465_MOESM1_ESM.docx]

**Supplemental material**

**Figure S1.** The surface morphology and elemental composition of ZnO-NPs were determined by scanning electron microscope (SEM-EDX).

**Figure S2.** Depiction of the pot experiment, showing wheat plants at four key growth stages: seedling, earing, filling, and maturity.

**Figure S3. Figure S3.** Effects of ZnO-NPs on vanadium (V) accumulation in grain (a), glume (b), stem (c), leaf (d), root (e), and soil (f) at the mature stage. Data are means ± standard error (SE, n = 5).

**Figure S4.** Effect of ZnO-NPs on the Zn content in the seed under V stress.

**Figure S5**. Correlation between amino acid content and plant properties. Heatmap shows Spearman’s correlation coefficients for each pairwise comparison of measured traits. * Significant correlation at *P* < 0.05 (two-tailed).

**Figure S1.** The surface morphology and elemental composition of ZnO-NPs were determined by scanning electron microscope (SEM-EDX).

**Figure S2**. Depiction of the pot experiment, showing wheat plants at four key growth stages: seedling, earing, filling, and maturity.

**Figure S3.** Effects of ZnO-NPs on vanadium (V) accumulation in grain (a), glume (b), stem (c), leaf (d), root (e), and soil (f) at the mature stage. Data are means ± standard error (SE, n = 5). Different uppercase letters indicate significant differences across ZnO-NPs levels within the same V level, and different lowercase letters indicate significant differences across V levels within the same ZnO-NPs level (two-way ANOVA, Tukey's HSD test, ****P* <0.001, ***P* < 0.01, **P* < 0.05). ZnO(0), ZnO(1), and ZnO(2) correspond to soil ZnO-NPs concentrations of 0, 100, and 200 mg/kg, respectively; V (0), V(1), and V(2) correspond to soil V concentrations of 0, 100, and 200 mg V kg^-1^, respectively.

**Figure S4**. Effect of ZnO-NPs on the Zn content in the seed under V stress

**Figure S5**. Correlation between amino acid profiles and plant physiological traits. Heatmap depicts Spearman’s correlation coefficients for pairwise comparisons among all measured traits. Asterisks denote statistically significant correlation (*P* < 0.05; two-tailed test).
